# Targeting sensory nerves in the tumor microenvironment: a new vulnerability in cancer therapy

**DOI:** 10.1186/s12964-025-02637-7

**Published:** 2026-01-29

**Authors:** Jun Jiang, Zhe Xu, Danyu Qu, Jiayang Qin, Weihong Wen, Weijun Qin, Donghui Han

**Affiliations:** 1 Department of Urology, Xijing Hospital, Air Force Medical University, Xi’an, China; 2 Department of Health Service, Base of Health Service, Air Force Medical University, Xi’an, China; 3 Department of Geriatric Medicine, Xijing Hospital, Air Force Medical University, Xi’an, China; 4 Medical School of Yan’an, Yan’an University, Yan’an, China; 5 West China School of Medicine, West China Hospital, Sichuan University, Chengdu, China; 6 Institute of Medical Research, Northwestern Polytechnical University, Xi’an, China

**Keywords:** Tumor innervation, Tumor microenvironment, Sensory nerve, Peripheral nervous system

## Abstract

Tumor innervation, the infiltration of nerves into the tumor microenvironment (TME), is increasingly recognized as a novel hallmark driving cancer progression and is associated with poor patient prognosis across various solid malignancies. This process is orchestrated by a complex, bidirectional crosstalk. Cancer and stromal cells release neurotrophic factors that induce axonogenesis or neurogenesis. In turn, the infiltrating nerves, particularly sensory nerves, secrete neurotransmitters, neuropeptides or form pseudo-synapse with tumor cells to facilitate cancer hallmarks, including sustained proliferation, invasion, metastasis, modulation of the anti-tumor immune response, and cancer plasticity. However, the specific contributions and underlying mechanisms of sensory nerve innervation in orchestrating malignancy remain incompletely elucidated. This review aims to synthesize the current understanding of the multifaceted roles of sensory neurons within the TME, detailing their intricate interactions with cancer and stromal cells, and highlighting the emerging therapeutic strategies that target the sensory nerve-tumor axis.

## Introduction

The expansion of nerves in the TME plays an important role in tumor progression [1]. Emerging evidence compellingly demonstrates that tumors co-opt and hijack these physiological nerve-dependent programs to promote their own growth, establishing a critical bidirectional crosstalk within the TME [2, 3]. The process by which the tumor recruits surrounding nerves into the TME via paracrine signals and subsequently enhances tumor growth is known as tumor innervation [4]. Intratumoral nerve density correlates with tumor malignancy, metastatic potential, recurrence rates, and poor clinical prognosis [5, 6]. This highlights the importance of tumor innervation as a critical tumor hallmark.

The impact of peripheral nervous system (PNS) on the TME has become a crucial factor in the pathogenesis of malignant tumors. PNS consists of three major functional types of nerves: sensory nerves, often identified by the marker calcitonin gene-related peptide (CGRP); sympathetic nerves, identified by tyrosine hydroxylase (TH); and parasympathetic nerves, identified by vesicular acetylcholine transporter (vACHT) [5, 7, 8]. Sympathetic and parasympathetic nerves primarily regulate the functional activities of organs such as the heart, blood vessels, abdominal viscera, smooth muscles, and glands. The predominant type of nerve innervation within a tumor varies considerably across different cancer types and is largely determined by the tumor’s tissue of origin. For example, sensory nerves preferentially infiltrate oral cavity squamous cell carcinoma (OSCC) [9], head and neck squamous cancer [10], and gastric cancer [11]. Similarly, in triple-negative breast cancer (TNBC), sensory nerve fibers are significantly more abundant than sympathetic counterparts [12]. Whereas prostate cancer is richly innervated by the autonomic nervous system, and inhibiting cholinergic nerve innervation by pharmacological blockade or genetic abrogation improved mouse survival [13].

**Fig. 1:**
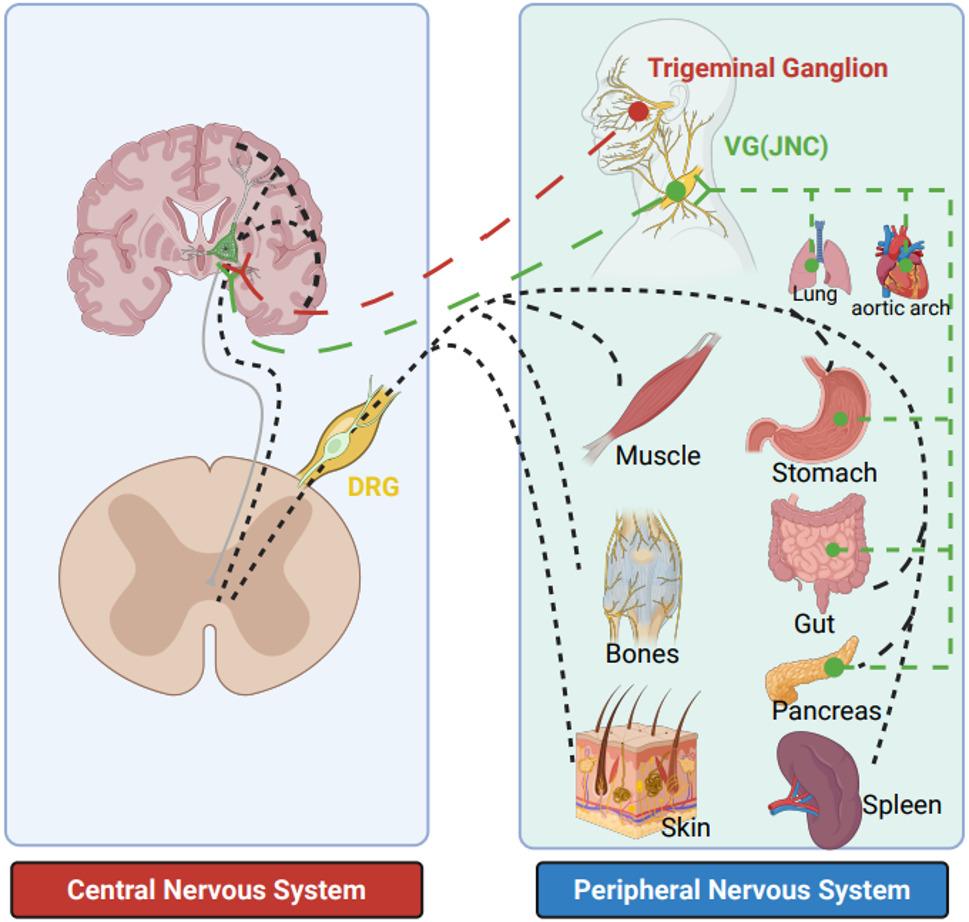
Peripheral sensory neurons diversity in DRGs, TG and JNC. Peripheral sensory neurons dominantly derived from DRGs, innervate skin, skeletal muscle and bones, as well as internal organs, including stomach [11], gut [16], pancreas [44, 58] and spleen [41]. Sensory nerves derived from JNC (via vagal nerve) primarily innervate internal organs, such as lung [20], aortic arch [22], pancreas [11], stomach [19] and gut [21]. Whereas sensory neurons derived from TG innervate head and meninges. DRGs, dorsal root ganglia; TG, Trigeminal ganglion; JNC, jugular-nodose ganglia

The intricate landscape of tumor innervation extends beyond the autonomic nervous system via adrenergic or cholinergic pathways [14, 15]. Sensory nerves, in particular, present a unique and complex area of study. Sensory nerves derived from dorsal root ganglia (DRG) along the spine, trigeminal ganglia (TG), and vagal ganglia (VG), are responsible for transmitting peripheral sensory information to the central nervous system (CNS) (Fig. 1). Sensory nerves derived from DRGs primarily innervate the skin, muscle, breast, as well as internal organs such as gut, stomach, and pancreas [16, 17]. Sensory nerves of TG mediate the somatic sensation of head and meninges [18], while sensory nerves of VG, with cell bodies located in jugular-nodose ganglia (JNC) primarily control breathing, blood pressure and gastrointestinal digestion [19–22]. Sensory nerves can be clustered based on their specific sensory modality they perceive, including mechanoreceptors, thermoreceptors, chemoreceptors, and nociceptors. The type of sensory nerve that is most closely associated with extracranial solid tumors and has been the focus of much research is the transient receptor potential vanilloid 1 (TRPV1, a ligand gated ion channel receptor) positive nociceptor, also known as the pain-sensing neuron [17, 23]. Nociceptor sensory nerves play a vital role in transmitting pain signals to CNS, and pre-treatment pain in cancer has been proven to be a poor prognostic marker [24, 25]. However, sensory nerves play a far more critical role in the occurrence and development of solid tumors than merely pain transmission. In this review, we summarize the specific mechanisms by which sensory neurons regulate the TME and describe how sensory nerves interact with the various components of the TME to promote cancer development and progression.

## Sensory innervation in the tumor microenvironment

**Fig. 2:**
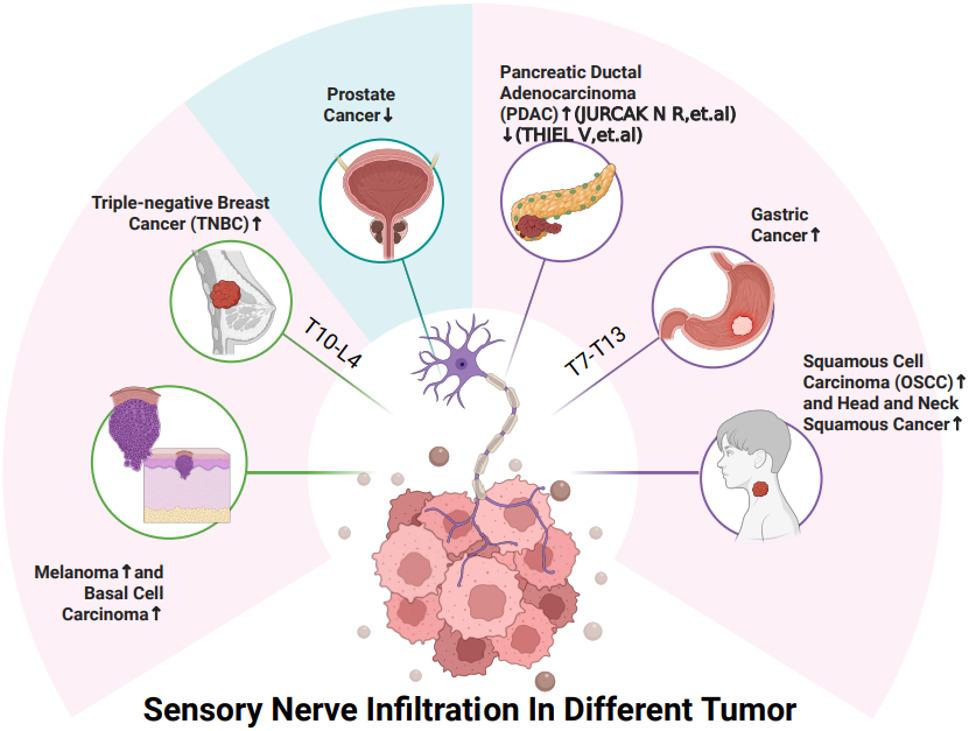
Peripheral sensory nerve infiltration density in different tumors. Abundant sensory nerve innervation is observed in numerous cancers, including TNBC [27], PDAC [44, 58], gastric cancer [11], melanoma and basal cell carcinoma [23, 30, 31], and OSCC and HNSC [9, 10, 32, 85]. The nerves innervating breast cancer derive from T10-L4 DRG sensory neurons, while those innervating gastric cancer derive from T7-T13 DRGs. In most of these malignancies, sensory nerves promote a pro-tumorigenic microenvironment and drive disease progression. In contrast, prostate cancer exhibits less sensory innervation, but with abundant sympathetic nerves infiltration [34]. *T* Thoracic, *L* Lumbosacral

Abundant sensory nerve innervation has been observed in multiple tumor models and patient pathological tissues, including in breast cancer [26, 27], pancreatic ductal adenocarcinoma (PDAC) [28, 29], gastric cancer [11], melanoma [23, 30, 31], head and neck cancer [10, 32], OSCC [9], and basal cell carcinoma [33]. In these cancers, sensory nerve infiltration promotes the transformation of various components of the TME towards a more malignant direction. In contrast, sensory nerve infiltration is reduced compared to normal tissue in prostate cancer [34] (Fig. 2). In breast invasive ductal carcinoma (IDC), nerve growth is associated with some invasive characteristics of IDC within the ER-negative and lymph node-negative subtypes [26]. Additionally, neural invasion is considered a high-risk factor for the malignancy of PDAC [35]. Notably, the impact of sensory nerve ablation on tumor growth might depend on the timing of the intervention. Intriguing findings in melanoma demonstrated ablation of sensory nerves with resiniferatoxin (RTX) before tumor implantation paradoxically accelerated tumor growth. Conversely, when the same RTX treatment was administered to mice with established tumors, it significantly inhibited tumor progression [31]. This highlights a potential dual role for sensory nerves, suggesting they may have distinct functions during the initial stages of tumor establishment versus the progression of an existing tumor. Collectively, these findings suggest that the degree of sensory nerve innervation strongly correlates with tumor aggressiveness and poor prognosis, particularly in established tumors.

### The origin of sensory nerve tumor innervation

Regarding the origin of intratumor peripheral sensory neurons, three primary hypotheses have been proposed. Firstly, most studies demonstrated that tumors can recruit or reprogram peripheral nerves from DRG, TG and VG to enter the TME and support tumor growth and metastasis [10, 36]. Secondly, intratumor neurons also can originate from the migration of neural progenitor cells expressing doublecortin from CNS [37], which are then reprogramed into sensory neurons by tumor cells. Finally, another hypothesis supports that tumor cells can lose their native lineage identity and dedifferentiate into neural progenitor cells with stem-like characteristics, without typical mature neuronal markers and neuroendocrine lineage markers [38]. Definitively distinguishing between these hypotheses requires sophisticated experimental approaches. Genetic lineage tracing is essential to unequivocally track the cellular origin, whether from pre-existing neurons, neural progenitors, or the tumor cells themselves. Furthermore, combining single-cell and spatial transcriptomics will be crucial to map the signaling networks that orchestrate this process and to identify any intermediate cellular states that might precede neurogenesis within the tumor.

### The perception of sensory neurons in TME

Sensory nerves innervating the tumor are not bystanders but act as sophisticated environmental sentinels, equipped to detect a complex chemical, physical, and biological stimuli (e.g., pH, heat, pain, mechanical and inflammatory stimuli) within the TME. A primary hallmark of the TME is profound local acidosis, resulting from the Warburg effect and hypoxic conditions, which are directly sensed by the dominating TRPV1-expressing nociceptor neurons. TRPV1 receptor is essential for tumor growth, and genetic ablation of the *TRPV1* gene, or pharmacological silencing of nociceptors decreased tumor growth [23]. Beyond pH, sensory afferents are constantly bathed in a tumor-promoting inflammatory TME rich in noxious stimuli. This mixture, released by both tumor and stromal cells, contains damage-associated molecular patterns (DAMPs) [39], prostaglandins (e.g., PGE₂) [40, 41], cytokines (e.g., TNF-α, IL-1β) [42, 43]. TRVP1-expressing nerves also express tropomyosin receptor kinase A (TrkA), which responds to nerve growth factor (NGF) released by cancer cell or stromal cell like cancer-associated fibroblasts (CAFs), facilitating their recruitment into the TME [44].

Beyond the well-characterized TRPV1-expressing sensory neuron, a distinct subset of PROKR2-positive sensory neurons has also been identified [45]. These neurons exhibit a highly specific innervation pattern, targeting the deep fascial tissues of the limbs while sparing the cutaneous epidermis and primary abdominal fascia. Notably, this population has been reported to exert anti-tumor effects in TNBC through stimulation of the vagal-adrenal pathway [46].

Concurrently, the TME presents significant mechanical stimuli, such as interstitial fluid pressure induced by leaky vasculature and stiffness derived from accumulation of extracellular matrix secreted by CAFs. These mechanical stresses are transduced by specialized mechanosensitive ion channels, notably the Piezo1 + and Piezo2 + sensory nerves from DRG [47, 48]. The integration of these diverse noxious signals by peripheral nerve endings represents the critical first step that initiates neurogenic contributions to cancer progression. To date, investigations into the role of sensory nerves in the TME have predominantly centered on their perception of inflammatory mediators and pain signals. However, there is a notable scarcity of literature concerning their ability to sense mechanical stimuli, suggesting that this area is a valuable and underexplored field for future studies.

### The subtypes of sensory nerve in tumors

Sensory nerves are not a monolithic entity but rather exhibit remarkable heterogeneity. Consequently, their classification system is multifaceted, traditionally relying on a combination of key features to define distinct subtypes. These features include their functional modality (nociceptors, mechanoreceptors, thermoreceptors), their unique neurochemical signature, such as the secretion of neuropeptides like CGRP and SP, and their fundamental biophysical properties. The latter, encompassing axon diameter and myelination, gives rise to the well-established classification of sensory afferents into fast-conducting A-fibers and slow-conducting C-fibers [49], which are critically involved in different physiological and pathological processes, including tumor progression. Recently, the advent of single-cell sequencing has identified 17 clusters of sensory nerve subtypes in the mouse DRG based on molecular markers, transcriptomic profiles, and functional properties [50].

Additionally, a neurochemical perspective, specifically examining the interplay between sensory afferents and the glutamatergic and GABAergic/enkephalinergic interneurons, is essential for maintaining host defense homeostasis. Sensory neurons are generally glutamatergic, serving as the primary source of the excitatory neurotransmitter glutamate. Their signaling, however, is exquisitely limited by the inhibitory GABAergic/enkephalinergic system, a regulatory mechanism that operates mainly at synapses within the dorsal spinal cord of the CNS [51]. The balance between excitatory and inhibitory signaling is crucial for controlling inputs of peripheral sensory afferents, especially in maintaining proper host defense mechanisms in pathological contexts such as cancer. For instance, GABAergic neurons originating from rostral ventromedial medulla (RVM) project to the spinal dorsal horn and specifically inhibit local inhibitory interneurons (e.g., GABAergic/enkephalinergic interneurons). By suppressing these spinal ‘gatekeepers’ of pain, the RVM effectively removes the brake on ascending nociceptive pathways, leading to an amplification of pain perception from peripheral sensory nerve afferents [52].

Nociceptor sensory nerves expressing TRPV1 are the most extensively investigated within the TME, are classically subdivided into two main subtypes, peptidergic (CGRP, SP, and galanin) and non-peptidergic (isolectin B4, IB4) subtypes [53]. Hyper-innervation by peptidergic sensory nerves has been documented in various extracranial solid tumors. For instance, compared to normal human stomach, diffuse-type GC exhibits higher CGRP + nerve density in gastric cancer (GC). Moreover, using different types of tracing viruses to mark nerves, it was found that all marked nerves expressed CGRP, indicating that GC cells preferentially attract CGRP + sensory nerves [11]. However, other peptidergic (not CGRP+/SP+) and non-peptidergic sensory nerve subtypes have not been extensively reported. A possible reason is that the existing research focuses on sensory nerve subtypes with higher proportions, and many nerve subtypes have only recently been identified and have lower proportions. Therefore, further research is urgently needed to discover their specific functions in the TME.

## Crosstalk between sensory nerves and components of the TME

### Sensory nerves crosstalk with tumor cells by paracrine mechanisms and synaptic contact

**Fig. 3:**
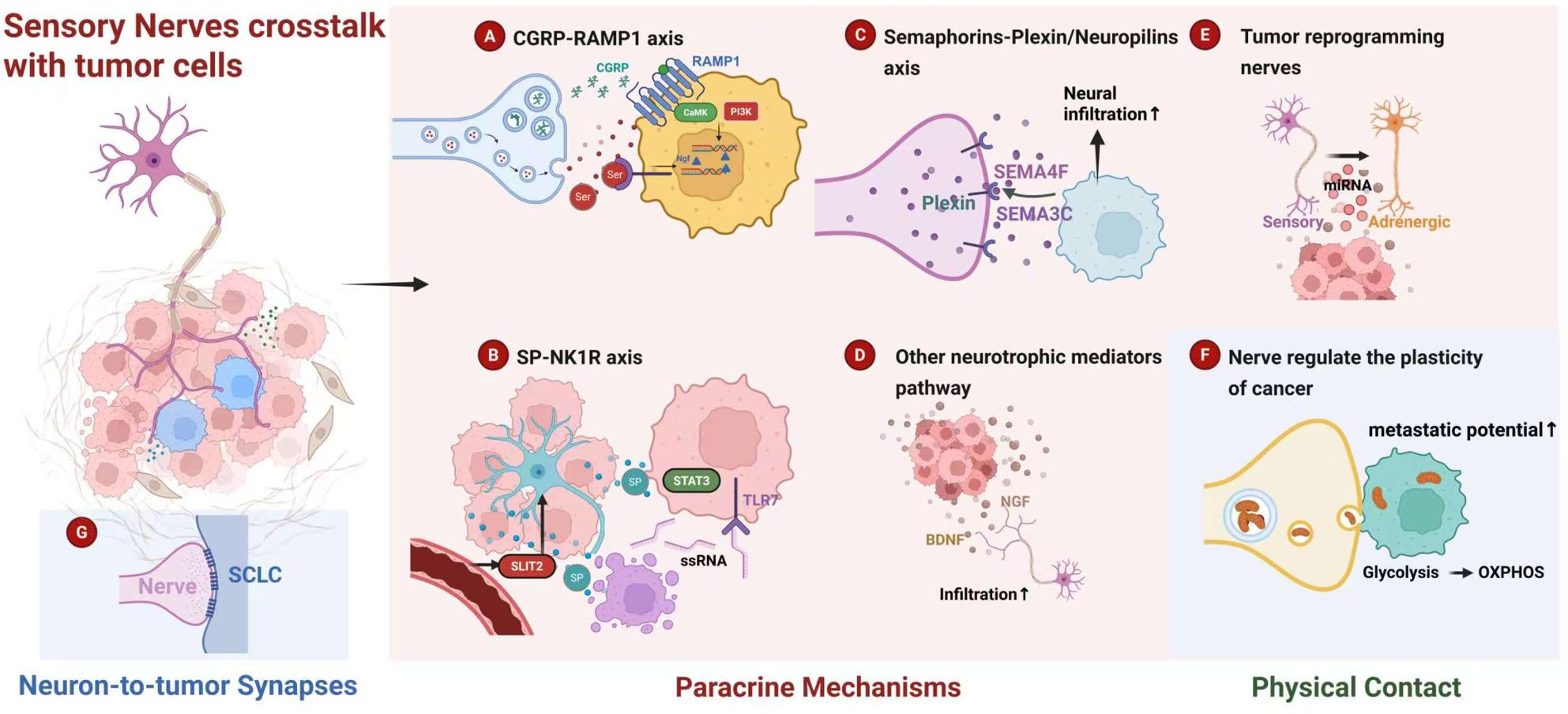
Crosstalk between tumor cells and sensory neurons by neurochemical paracrine mechanisms, pseudo-synapse and physical contact. **a** Activated sensory neurons release CGRP binds to RAMP1 receptor expressed on tumor cells to promote cancer progression. **b** SLIT2 derived from the tumor vascular endothelium promotes sensory nerve innervation, leading to the release of Substance P (SP). SP induces apoptosis in a small subpopulation of cancer cells, which releases ssRNA. Subsequently, ssRNA activates surviving, TLR7-expressing tumor cells to drive overall tumor growth and metastasis [27]. **c** Tumor expressing semaphorins elicit sensory nerve infiltrate into TME by binding to Plexin/Neuropilins receptors. **d** Cancer cells secrete TGF-α [64], NGF [66] or BDNF [67] to recruit and nourish sensory neurons. **e** Sensory nerves can be reprogramed into adrenergic neurons [10]. **f** Mitochondria derived from neurons can be transferred into tumor cells via directly physical connection [68]. **g** Vagal nerves stimulate SCLC tumorigenesis and progression via pseudo-synapse [54, 55]. *ssRNA* Single-stranded RNA, *TGF-α* Transforming growth factor α, *NGF* Nerve growth factor, *BDNF* Brain-derived neurotrophic factor

Sensory nerves are involved in multiple stages of tumor progression, mainly through paracrine signaling from neurons to tumor. Nerves can also form tumor-to-neuron synapses, which have been reported in intracranial tumor glioma, as well as in the peripheral tumor small cell lung cancer (SCLC) which possess neuroendocrine characteristics [54, 55] (Fig. 3). Data from both mouse model of SCLC and human SCLC samples support an abundance of neuron-like tumor synapses and, in particular, glutamatergic signaling within the TME. Besides direct physical contact, noxious stimuli from the TME activate TRPV1-sensory nerves to secrete neuropeptides and other proteins, such as CGRP, SP, and galanin. These molecules then transmit chemical signals that promote the malignant properties of tumors.

#### CGRP-RAMP1 signaling

CGRP, encoded by *Calca* gene, is one of the most well-known of neuropeptide, and released by nociceptive sensory nerves after TRPV1 activation. The CGRP-RAMP1 signaling pathway is considered a pivotal pathway for nerve-tumor crosstalk. The binding of CGRP to RAMP1 leads to multiple downstream signaling pathways, including the PI3K-AKT and CaMK pathways [56]. Wang et al. reported that Rb-E2F pathway was downstream of CGRP-RAMP1 activation, and wortmannin and KN-93 kinase inhibitor significantly suppressed CGRP-dependent E2F activity [11].

#### SP-NK1R signaling

Sensory nerves are involved in the regulation of tumor occurrence and development at various stages. Immunohistochemical staining shows the presence of the neuropeptide substance P (SP) in the tumor interstitium. SP, released by sensory nerves within the TME, primarily acts by binding to its main receptor, the neurokinin 1 receptor (NK1R, encoded by TACR1), and subsequently activates key molecules in the downstream mitogen-activated protein (MAP) kinase pathway, such as ERK1/2 [57]. SP-NK1R axis has been shown to enhance proliferative and migratory capabilities in multiple cancer types, including breast cancer [27], pancreatic cancer [58], and prostate cancer [59].

SP was elevated significantly in breast cancer, particularly with a higher probability of lymph node metastasis [27]. SLIT2 specifically secreted by tumor endothelial cells drives the infiltration of sensory nerves (T10-L4) into TME. Sensory nerves further release SP, and increased cancer cell growth and migration, and pre-treatment with NK1R antagonist could inhibit this phenomenon. Mechanistically, the release of SP can drive a small group of NK1R + cancer cells to apoptosis. The apoptotic cells release single-stranded RNA (ssRNA), which activates Toll-like receptor 7 (TLR7) on the neighboring tumor cells to enhance metastasis by a MyD88-independent PI3K signaling [27].

In addition, a unique but minor subpopulation of neuroendocrine pancreatic intraepithelial neoplasms (PanIN) cells promote tumor progression via cross-talking with sensory nerves [58]. Mechanistically, SP derived from sensory nerves binding to NK1R expressed on neuroendocrine carcinoma promote their proliferation via activating the STAT3 pathway. Sensory denervation of a PDAC model by injecting TRPV1 antagonist capsaicin, impaired tumor progression along with loss of STAT activation [60].

In prostate cancer, SP-NK1R axis has been shown to promote cancer progression by modulating the cell cycle (c-Myc, cyclin D1, p21) and apoptosis (Bcl-2, Bax) [59]. Furthermore, this pathway promotes cell migration by upregulating the expression and activity of matrix metalloproteinases MMP-2 and MMP-9. Importantly, these pro-tumorigenic effects were demonstrated to be significantly reversed by the administration of the NK1R antagonist aprepitant [59].

#### Semaphorins-plexin/neuropilins signaling

The Semaphorin-Plexin/Neuropilins signaling axis, a key regulator of axon guidance, plays a pivotal role in orchestrating tumor innervation. This role was first characterized in prostate cancer, where tumor cells were found to hijack this pathway. By expressing molecules such as SEMA4F and SEMA3C-PlexinA2/NRP1, prostate cancer cells actively recruit neural infiltration [61, 62]. This induced innervation, stemming from the differentiation of local neuronal progenitors, contributes to a denser nerve network within the TME and correlates with heightened disease aggressiveness. Subsequent investigations have confirmed that this semaphorins-driven mechanism is not unique to prostate cancer; its pro-tumorigenic role has been validated in other malignancies, including breast [12] and pancreatic cancer [29].

TNBC is highly innervated dominantly by sensory nerves [12, 63]. DRG neurons co-culture with MDA-MB-231 cell promoted tumor migration by upregulating Plexin B3 expression on tumor. Plexin B3’s high affinity ligand, SEMA5A, is expressed in sensory nerves not cancer cells. SEMA5A-Plexin B3 axis mediates sensory nerve-driven TNBC metastasis. Additionally, direct co-culture of neurons with tumor cells accelerates cancer cell migration more significantly than culturing them with neuron-conditioned media alone. This suggests that tumor-to-neuron physical contact plays a more critical role in promoting cancer migration compared to secreted factors.

In PDAC samples from patients with perineural invasion (PNI), elevated levels of SEMA3D and Plexin D1 can be detected. Mechanistic studies have found that tumor cells expressing SEMA3D can move towards neurons expressing Plexin D1 in vitro. Furthermore, in vivo studies demonstrated that genetic ablation of Plexin D1 decreased intratumoral nerve density and metastasis in a murine model of PDAC. Within TME, DRG nerves induce pancreatic cancer cells to release SEMA3D. As an extracellular signal, SEMA3D activates its receptor Plexin D1 expressed on neurons, which in turn drives the migratory and invasive phenotype of pancreatic cancer cells [29].

#### Other neurogenic signaling

Highly expressed signaling molecules in the TME prompt sensory nerves to secrete neurotrophic or neurogenic factors, including TNF-α, vascular endothelial growth factor (VEGF), nerve growth factor (NGF) and brain-derived neurotrophic factor (BDNF). For example, in PDAC, the high expression of TGF-α by tumor cells promotes the secretion of CCL2 by sensory nerves, which in turn induces the over-phosphorylation of the cytoskeletal protein paxillin in cancer cells through CCR4, further enhances tumor metastasis [64]. CCR4 antagonist C021 or paxillin inhibitor 6-B345TTQ treatment reduced PNI density and tumor burden [64].

VEGF and NGF are involved in axonogenesis in breast cancer. In a rat model of breast cancer, it was found that the density of sensory nerves increased with tumor growth, and VEGFR inhibitor SU5416, NGFR inhibitor AG879 decreased both the nerve density and vascular density of breast tumor [65]. In PDAC, peripheral axons release serine (Ser) to provide exogenous Ser (exSer) for PDAC cells. Peripheral nerve axons can rescue the growth of exSer-dependent PDAC cells in exSer-deprived mice, and in turn, PDAC cells selectively translate and secrete nerve growth factor (NGF) to promote tumor innervation [66]. Similarly, NGF knockout or TRK inhibitor entrectinib suppressed CGRP + sensory nerve innervation in gastric cancer, indicating sensory neurons expansions is dependent on NGF/TRK signaling [11].

BDNF was observed to be highly expressed in OSCC and in close proximity to sensory nerve, and BDNF blockade with TrkB-Chimera Ab or TrkB (BDNF receptor) inhibitor ANA12 reverses tongue tumor-induced pain behaviors [67]. Tumor cells can also influence sensory nerve innervation through the secretion of exosomes in head and neck squamous cell carcinomas. It was found that tumor cell-derived exosomes promote the innervation of the pheochromocytoma cell line PC12, and exosomes containing EphrinB1 further enhance neurite outgrowth in PC12. Inhibition exosome release by genetically knockout Rab27A and Rab27B, two small GTPases contributing to exosome release, attenuates innervation in vivo [8]. Together, tumor cells influence sensory nerves through direct chemical communication, secretion of neurotrophic factors, and extracellular vesicles, thereby promoting the occurrence of abnormal tumor innervation.

#### Tumor plasticity reprogrammed by nerve-to-cancer transfer of mitochondria

The intercellular transfer of mitochondria, once documented primarily between tumor and immune cells, is now recognized as a key feature of nerve-tumor interactions. Employing a novel MitoTRACER system, Grelet et al. demonstrated that nerves establish direct physical contact with tumor cells via tunneling nanotube-like structures, which serve as conduits for organelle transfer [68] (Table 1). Through this process, nerves donate their mitochondria to cancer cells, leading to a notable metabolic reprogramming in the recipient cells. This is characterized by a hallmark shift from a predominantly glycolytic phenotype to one reliant on oxidative phosphorylation (OXPHOS) [68]. Furthermore, this study found that denervation with botulinum neurotoxin type A (BoNT/A) impaired tumor malignancy. The functional significance of this mitochondrial transfer was confirmed in in vivo models of breast cancer and melanoma, where tumor cells that had assimilated neuronal mitochondria exhibited enhanced metastatic potential and a distinct tropism for the brain [68].

**Table 1:** Summary of denervation approach in different cancers

Cancer	Tumor-bearing Mouse Model	Denervation	Crosstalk	Mechanism	Ref.
GC	Atp4b-cre; Cdh1^fl/fl^;Kras^G12D^;Trp53^fl/fl^;YFP mice, Cck2r-creERT; Kras^G12D^	Calca-cre, Trpv1-cre, Trki (entrectinib), NGF-KO, Rimegepant; RAMP1-KO	CGRP-RAMP1 axis, NGF-TrkA axis	Paracrine	[11]
TNBC	MMTV-PyMT;	Tac1-KO, Nav1.8-cre, aprepitant	SP-NK1R axis	Paracrine	[27]
PDAC	KPC cells orthotopic injection	RTX, Trpv1-DTR, Trpv1-Cre	CGRP-RAMP1 axis; NGF	Paracrine	[44]
TNBC	4T1 mammary fat pad injection, Intraductal human-in-mouse transplantation model	BoNT/A	mitochondria	Physical contact	[68]
SCLC	Rb1^fl/fl^Trp53^fl/fl^Rbl2^fl/fl^, Rb1^fl/fl^Trp53^fl/fl^	Synaptic genes piggyBac insertional mutagenesis screen in vivo, DCPG, Riluzole	Glutamate/GABA	Pseudo-synapse	[89]
SCLC	Rb1^fl/fl^Trp53^fl/fl^p130^fl/fl^	Vagus nerve transection, levetiracetam	Glutamate/GABA	Pseudo-synapse	[90]
PDAC	KPC cells Orthotopic injection	Capsaicin, Tpv1-KO, Grin2D^−/−^	Glutamate-NMDA axis	Pseudo-synapse	[91]

*SCLC* Small Cell Lung Cancer, *PDAC * Pancreatic Ductal Adenocarcinoma, *TNBC * Triple-Negative Breast Cancer, *GC * Gastric Cancer

However, the identity of the specific nerve type acting as the mitochondrial donor remains a critical unanswered question. The foundational experiments by Grelet et al. utilized co-cultures with CNS-derived neurons, which may not fully recapitulate the peripheral tumor microenvironment. An important clue may lie in the observation that BoNT/A suppresses breast cancer metastasis. Since the established mechanism of BoNT/A involves cleaving SNAP-25 to inhibit neuropeptide release (e.g., CGRP, SP) from sensory nerves [69]. Given the dense sensory innervation of TNBC validated by numerous literatures [12, 27, 70], it is plausible to hypothesize that the therapeutic effect of BoNT/A arising from disrupting a pro-metastatic interaction with this specific nerve subtype.

#### Sensory nerve plasticity tamed by cancer cell

Lineage plasticity, a known feature of tumor cells, is also observed in intra-tumoral nervous system. The sensory nerve types composition within the TME is not fixed; for instance, infiltrating sensory nerves can be reprogrammed by cancer cells to transform into other subtypes, even to adrenergic nerves. Pancreatic cancer is well-known for its dense sensory hyper-innervation [44, 58], however, Trumpp et al. have demonstrated that these CGRP + sensory nerves could be converted into SLIT2 + sensory nerves, and even reprogramed into TH + sympathetic nerves [5]. Single-cell sequencing results of PDAC revealed that the number of sympathetic neurons (TH+) in PDAC model was 2.3 times higher than in healthy tissue, while the distribution density of sensory nerves (CGRP+) decreased by 62%. This shift in nerve subtypes allows tumor to acquire more growth factors (such as SLIT2, which promotes angiogenesis) and evade immune surveillance.

Additionally, in a head and neck tumor model, the absence of TP53 led to the adrenergic transdifferentiation of tumor-associated sensory nerves. It was found that cancer-derived extracellular vesicle (EV), containing key components such as miR-21, miR-34a, or miR-324, induce several transcription factors (Ntrk1 and Isl1, etc.) crucial for nerve cell fate and catecholaminergic differentiation, promoting the adrenergic differentiation of cancer-associated nerves [10].

### Sensory nerves and CAFs

**Fig. 4:**
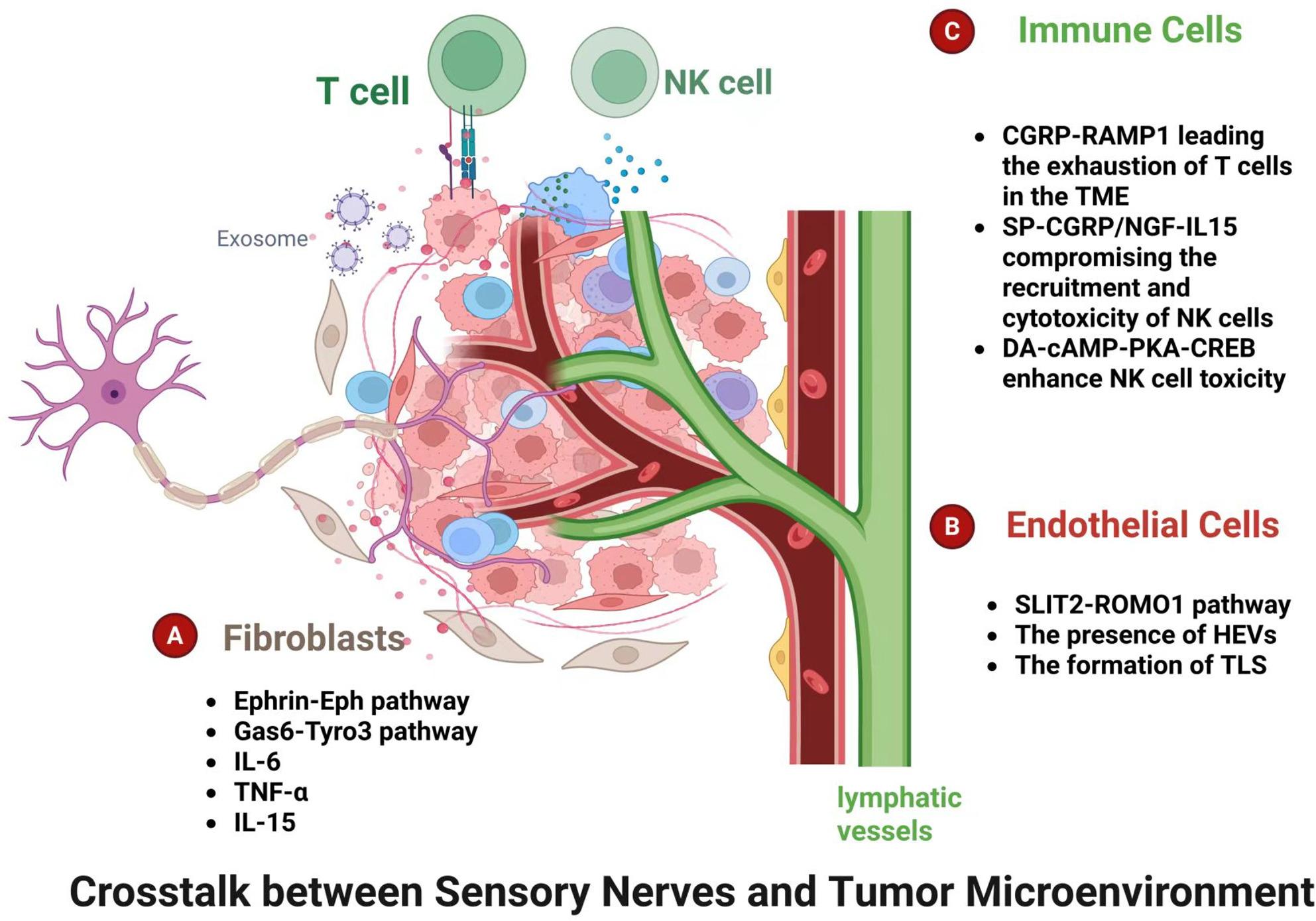
Crosstalk between sensory nerves and major stromal components. The sensory afferents are potent modulators of the TME, perceiving physicochemical changes such as pH, heat, inflammatory molecules and stiffness. Upon stimulation, they release a spectrum of neuropeptides. **a** These neuro-mediators profoundly remodel the TME by modulating the function of CAF, **c** T cells, NK cells and **b** endothelial cells, fostering an immunosuppressive TME. This dynamic reprograming circuit sustain malignant progression and contributing to therapeutic resistance

Analysis of single-cell data from the TME revealed that the correlation between tumor cells and CAFs increased by 2.8 times in PDAC. Key signaling pathways include the Ephrin-Eph pathway, which promotes the physical connection between nerves and CAFs, and the Gas6-Tyro3 pathway, which activates tumor cell proliferation (Fig. 4). When researchers co-cultured neurons with CAFs in vitro, the proliferation rate of CAFs increased by 50%, and they secreted more pro-inflammatory factors such as IL-6 and TNF-α, creating a vicious cycle [5]. In the gastric cancer model, nociceptive nerves promote the growth of GC spheroids in a concentration-dependent manner through CGRP, enhancing the in vitro spheroid formation of GC cells. Notably, chemogenetic activation of nociceptive nerves led to an increase in CAFs in gastric tumors. CGRP maintains the proliferation of CAFs in a concentration-dependent manner, and CGRP treatment significantly increased IL-6 in CAFs [11]. Similarly, in the intratumoral innervation of PDAC, CGRP and NGF interact with CAFs [71]. In vitro, co-culture and ELISA assays showed that this interaction led to the inhibition of IL-15 expression in CAFs, and flow cytometry assay demonstrated interaction of nociceptive nerves with CAFs suppressed cytotoxic function of natural killer (NK) cells by reducing IL-15 level. NGF*-*KO CAFs reduced the CGRP production of nociceptive nerves. However, there is a notable lack of in vivo studies to validate these interaction mechanisms and to definitively determine their functional impact on tumor growth. In sum, when sensory nerve innervation increases in tumors, it often leads to a dramatic increase in the secretion of inflammatory factors such as IL-6 and IL-15, causing the proliferation of CAFs and promoting the malignancy of cancer.

### Sensory nerves and endothelial cells

Sensory nerve innervation is closely related to the formation of blood vessels and lymphatics within tumors. Elevated expression of the axon guidance molecule SLIT2 has been demonstrated within the vascular endothelium of highly metastatic breast tumors [72]. Critically, targeted ablation of SLIT2 specifically in endothelial cells led to a significant reduction in both overall and sensory nerve density. In contrast, stromal deletion of SLIT2 failed to replicate this phenotype, providing compelling evidence that endothelium-derived SLIT2 is a pivotal mediator orchestrating breast tumor innervation [27]. Interestingly, SLIT2 derived from tumor cell exerts an opposite effect in PDAC which inhibits neural invasion and metastasis [73]. While SLIT2 expression is minimal in PDAC tumor cells, in vivo studies have shown that its exogenous overexpression potently suppresses tumor growth and peritoneal metastasis. This anti-tumor effect, mediated through binding to its receptor ROBO1, also includes the inhibition of angiogenesis and neural infiltration (Fig. 4).

High endothelial venules (HEVs) are specialized post-capillary venules that function as the primary gateways for circulating naïve and central memory lymphocytes (T cells and B cells) to enter into lymphoid tissues from the bloodstream [74]. The presence of HEVs correlates with good clinical characteristics, particularly in melanoma [75]. The formation of HEVs is an indispensable feature of mature, functional tertiary lymphoid structures (TLS) within tumors. TLS is an ectopic lymph node-like structure containing variable tissue B cell and T cell aggregates surrounding lymph and HEVs characterized by peripheral node addressin (PNAd) and the vascular addressin MECA79 [76]. Interestingly, cutaneous sensory nerves have been demonstrated to impede the maturation of intratumoral HEVs and restrict the formation of classical TLS in melanoma. The formation of TLS within melanoma is considered an indicator of sensitivity to immunotherapy. In contrast, when cutaneous sensory innervation was removed, TLS maturation was not limited, and denervation further increased T cell expansion and enhance anti-tumoral effect [30]. However, the specific molecular mechanisms underlying the inhibition of TLS maturation by sensory innervation remain to be elucidated.

### Sensory nerves and Schwann cells

Schwann cells (SCs), glial cells in the PNS, are key neural components in the TME that protect and nourish sensory nerve by forming myelin sheath and enveloping axon of neurons [77]. Additionally, SCs are high plastic cells in response to nerve injury and reprogramed by c-Jun, Notch and SOX2 signaling, which are implicated in driving PNI [78, 79]. Wong et al. reported that SCs envelop sensory axons can provide a track for tumor invasion. In PDAC, tumor cells can activate the transcription factor c-Jun to reprogram SCs into a non-myelinated/repair cell differentiation state. At this time, SCs reorganize cancer cells into chains and create migration trajectories, forming tumor-activated schwann cell tracks (TAST), which play an important role in tumor migration [80].

### Sensory nerves and immune cells

Neuro-immune interactions in TME are increasingly recognized as major drivers of tumor development and progression [81]. Sensory nerve infiltration primarily regulates tumor immunity by modulating the function of tumor-killing cells (T cells, NK cells, etc.) (Fig. 4). The expression of receptors for sensory neuropeptides, CGRP and SP, is not restricted to tumor cells, also expressed on immune cell populations, establishing a direct neuro-immune circuits. The activity of T cells is highly correlated with sensory nerve infiltration within the tumor. For example, a scRNA-seq analysis in melanoma demonstrates CGRP increases the exhaustion of RAMP1 + CD8 + T cells in the TME. As a result, RAMP1 + CD8 + T cells were more exhausted than their RAMP1-negative counterparts [23]. Mechanistically, the expression of cytotoxic molecules (e.g., IFNγ and TNF) and proliferative capacity (IL-2) of CD8^+^ T cells is lost, while the expression of exhaustion markers (PD-1 + LAG3 + TIM3+) of CD8^+^ T cells increases, reducing their ability to eliminate tumor cells [23]. Nevertheless, sensory nerve denervation via *TPRV1* genetic ablation and RAMP1 antagonism both reduce the CD8 + T cell exhaustion. Additionally, sensory nerve ablation also increases T cell clonality, expands the B cell repertoire, stimulates the formation of TLS-like structures and develops the protective anti-tumor immune responses in the TME [30].

Sensory nerves can also inhibit the infiltration and cytotoxic function of natural killer (NK) cells, thereby promoting the progression of PDAC [71]. Within the PDAC TME, nociceptive nerves engage in crosstalk with CAFs and NK cells, in which nerves release CGRP and NGF to downregulate IL-15 expression of CAFs, subsequently compromising both the recruitment and cytotoxicity of NK cells and fostering an immunosuppressive landscape. This cascade ultimately accelerates tumor progression and exacerbates the severity of cancer-associated pain. Ablation of nociceptor nerves by RTX markedly reduces tumor burden and prolongs overall survival time in an in vivo model. Additionally, clinical evidence from PDAC cohorts demonstrated that the extent of nociceptive innervation was inversely proportional to NK cell abundance while being directly proportional to reported pain intensity.

Increasing studies demonstrate that stimulating sciatic nerve sensory afferents at the ST36 point systemically modulates immune function through the vagal-adrenal reflex [45, 82]. The response rates to immunotherapy in TNBC have been disappointingly low, ranging from 5% to 23% [83, 84]. Recently, Ma et al. reported that combining sciatic nerve stimulation with anti-PD-1 therapy is feasible to overcome immune resistance in TNBC [46]. When sensory fibers of the sciatic nerve is stimulated, NK and cytotoxic CD8 + T cells in the TME are in an activated state. Mechanistically, stimulation of PROKR2 + sensory nerves activates the vagal-adrenal axis, releasing dopamine (DA), which activates the D1-like dopamine receptor-cAMP-PKA-CREB signaling pathway to enhance NK cell toxicity and inhibit tumor growth [46]. In addition, the combination of sciatic nerve stimulation and PD-L1 inhibition has been shown to yield synergistic anti-tumor efficacy in TNBC.

A recent study in HNSCC revealed that high nociceptive sensory nerve innervation and associated pain correlate with a significant increase in M2-like TAMs, with spatial analysis confirming their direct interaction [85]. Mechanistically, TAM-proximal cancer cells activate trigeminal ganglion-derived axons via the ATF4-SLIT2 axis, promoting axonogenesis. These activated nerves, in turn, remotely remodel cervical tumor-draining lymph nodes (TDLNs) to promote M2 polarization via the CCR5/CCR1 axis. Targeting this ATF4-SLIT2-CGRP axis, restores anti-tumor immunity, alleviates pain, and enhances the efficacy of immune checkpoint blockade. Notably, the RAMP1 antagonist rimegepant (used for migraine treatment) demonstrated this dual therapeutic benefit in preclinical models. Therefore, sensory nerves are revealed to be fundamental modulators of anti-tumor response, exerting pivotal roles in reprogramming crucial immune populations like NK cells, CD8 + T cells, and TAMs within the TME.

## Anti-sensory nerve innervation therapy

**Fig. 5:**
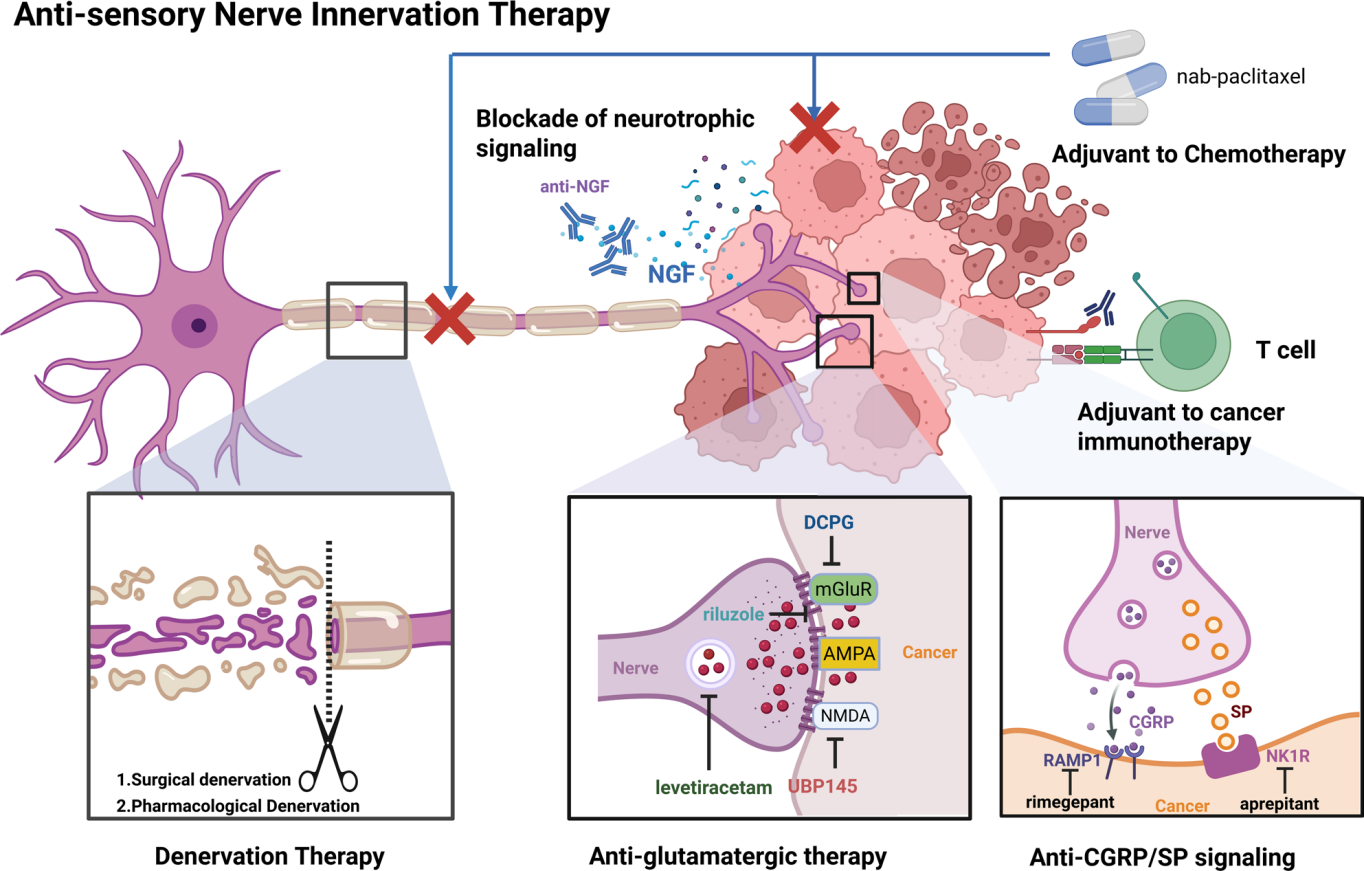
Anti-sensory Nerve Innervation Therapy. The pro-tumoral effects of sensory innervation can be therapeutically targeted at multiple levels. Key strategies depicted include complete denervation (surgical/pharmacological), blockade of neurotrophic mediators derived from tumor or stromal cells (e.g., anti-NGF), disruption of synaptic contact via anti-glutamatergic drugs including inhibiting glutamate release (levetiracetam [90] and riluzole [89]) and glutamate receptor activation (DCPG [89] and UBP145 [91]), inhibition of paracrine effect like RAMP1 and NK1R antagonist (rimegepant [44, 85] and aprepitant [27], respectively). By inhibiting this neuro-tumor axis, these approaches can potentiate the anti-cancer activity of standard chemotherapy and immunotherapy

Targeting sensory nerve activity is emerging as a promising anti-cancer strategy [86]. The primary approach involves the direct removal of sensory nerves innervating the tumor, a simple and effective treatment modality. Interventions can be designed to disrupt the multifaceted interactions between sensory nerves and the TME by targeting pivotal points within this communication network (Fig. 5). Moreover, there is compelling evidence that integrating nerve inhibition with existing anti-tumor treatments can significantly potentiate their effects, leading to a more robust therapeutic response.

### Denervation therapy

Preclinical studies have employed surgical denervation (neurectomy) or chemical ablation to eliminate nociceptive nerves, therefore inhibiting tumor progression. Surgical denervation of dorsal sensory cutaneous fibers, offers the advantage of complete and permanent removal of sensory nerves. It has been demonstrated that surgical denervation of dorsal thoracic sensory cutaneous fibers notably slows melanoma growth [30]. However, the procedure is technically demanding, highly invasive, and is often associated with substantial post-operative morbidity. In contrast, the chemical ablation approach offers a more refined and clinically relevant alternative. Neurotoxins such as RTX and capsaicin, which selectively target the TRPV1 channel, irreversibly cause functional desensitization and excitotoxic death of sensory neurons. Building on this evidence, TRPV1 has emerged as a promising therapeutic target in multiple cancer types. Accordingly, RTX has advanced to clinical trials for the management of cancer-related pain [87].

Pharmacological or genetic intervening in sensory nerve-tumor crosstalk exerts anti-tumor effects for tumor growth, migration, and tumor immunity. However, in contrast to β2-adrenergic receptor antagonists, which have advanced to clinical trials [1], inhibitors targeting sensory nerve receptors remain confined to the preclinical stage. For instance, studies have shown that blocking with an NGF receptor inhibitor (e.g. TRK inhibitor, LOXO-101) reduces the growth of PDAC in an orthotopic mouse model [66]. During the metastasis of breast cancer, blocking the communication between the neuropeptide SP and NK1R/TACR1 with a TACR1 antagonist, the antiemetic drug aprepitant, significantly inhibited TNBC growth and metastasis both in an organoids mouse model and a PDX model [27]. Additionally, it was found that orally administered rimegepant can significantly inhibit the tumor burden and prolong survival in an orthotopic PDAC mouse model [44]. Since CGRP + sensory nerves themselves function as pain-sensing neurons, RAMP1 antagonists also have the effect of relieving cancer pain [44].

Finally, genetic manipulation in preclinical models, such as Cre-lox mediated gene deletion (e.g., *NGF* [11], *CGRP* [44], *TRPV1* [23], *TACR1* [27] and *RAMP1* [11])(Table 1), also provides a promising strategy for abrogating neuron-tumor crosstalk to inhibit tumorigenesis and progression.

While targeting tumor-infiltrating nerves presents a promising therapeutic avenue, a critical challenge lies in mitigating the potential on-target, off-tumor side effects. Surgical denervation, the most direct approach, is often impractical due to its high invasiveness, potential for permanent functional loss, and associated morbidity. Pharmacological blockade, while less invasive, carries its own risks stemming from systemic exposure. For instance, systemic inhibition of neural signals, though preclinically effective, has been linked to adverse events including peripheral paresthesia, focal mononeuropathies, and affective dysregulation [88].

### Anti-glutamatergic therapy

Previous studies have proposed that ASCL1 + and Neurod1 + SCLC subtypes with high neuroendocrine signature were enriched with genes expressed in the GO term ‘glutamatergic synapse’. In addition, the functional synapses between SCLC cells and glutamatergic neurons in vitro and in vivo had been both validated, suggesting that neuroendocrine lineage might benefit from glutamatergic sensory neurons [54]. In preclinical animal models, this was further validated by the significant reduction in SCLC tumor volume following treatment with anti-glutamatergic drugs riluzole and DCPG in Rb1/Trp53 double KO mouse model [89] (Table 1). Intriguingly, only riluzole, but not DCPG, when combined with etoposide and cisplatin, results in improved response and survival [89] (Table 2). These findings suggest that broadly suppressing presynaptic glutamate release, rather than blocking specific postsynaptic receptors, may be crucial for achieving synergy with platinum-based chemotherapy and overcoming resistance in SCLC.

**Table 2:** Denervation combining with chemotherapy

Cancer	Denervation	Chemotherapy	Model	Outcome	Ref.
SCLC	Riluzole	etoposide and cisplatin	Rb1^fl/fl^; Trp53^fl/fl^ mouse model	Improved survival	[89]
	DCPG			No improved response	
PDAC	6OHDA	nab-paclitaxel	PDX	Synergistic inhibition of tumor growth	[5]
		oxaliplatin		No synergistic inhibition	

Riluzole, inhibits glutamate release; DCPG, dicarboxyphenylglycine, mGluR2 antagonist; 6OHDA, target sympathetic neuron; nab-paclitaxel, target tumor cell and sensory axon

Additionally, a back-to-back study also reveals vagotomy at an early stage significantly inhibits lung tumor growth, liver metastasis, and prolong mouse survival, but it failed to affect advanced tumors, indicating the vagus nerve’s primary role is in tumor initiation [90]. Mechanistically, SCLC brain metastasis cells form functional synapses with neurons to receive glutamatergic and GABAergic signals (Table 1). Electrophysiology confirmed that these neural signals, particularly GABA, induce depolarization and promote SCLC proliferation. Optogenetics further proved this link, as either neuronal activation or direct depolarization of SCLC cells markedly enhanced tumor growth and invasion. Treatment with levetiracetam, a commonly used anti-seizure drug interfering with synaptic vesicle release, significantly inhibits tumor growth in a SCLC brain metastasis model in vivo [90].

The rationale for anti-glutamatergic therapy in solid tumors is powerfully reinforced by the recent discovery of functional ‘pseudo-synapses’ between sensory nerves and PDAC [91] (Table 1). This study demonstrated that sensory nerve-cancer junctions are selectively enriched with the NMDA receptor subunit GRIN2D/GluN2D, enabling tumor cells to directly respond to neuron-derived glutamate. This signaling was shown to drive proliferation and invasion, in part via the CAMK IV-CREB pathway. Crucially, disrupting this glutamate-GRIN2D axis markedly improved survival in preclinical PDAC models, thus validating this specific neuro-tumoral communication pathway as a high-value therapeutic target.

### Adjuvant to chemotherapy

Combined chemotherapy is the standard regimen for multiple cancers. Chemotherapy, like nab-paclitaxel, a taxane, disrupts microtubule function, which simultaneously arrests the cell cycle in tumor cells and preferentially impairs axonal transport in highly dependent sensory nerves. While this neurotoxicity, so-called peripheral neuropathy, manifests as a common adverse effect of taxanes, it paradoxically predicts better patient outcomes [92, 93]. Therefore, denervation with 6OHDA (sympathetic neuron ablation) as an adjunct to nab-paclitaxel chemotherapy (targeting both tumor cell and sensory nerves) can achieve synergistic inhibition of tumor growth [5] (Table 1). Such a combination approach not only exerts direct cytotoxicity on tumor cells but also disrupts intratumoral neural connections. For example, the combination of paclitaxel and denervation therapy reduced the PDAC tumor size by 16.5 times, far exceeding the effect of monotherapy [5]. However, 6OHDA combined with another standard chemotherapy for PDAC, oxaliplatin, does not result in a synergistic inhibition of tumor burden. These results indicates that nab-paclitaxel enhances anti-tumor effects by inhibiting sensory nerve axon outgrowth, an effect not observed with oxaliplatin. This provides a mechanistic explanation for the unique clinical efficacy of taxanes. Paclitaxel-induced peripheral neuropathy often manifests as chronic pain, burning, and tingling in the hands and feet, which is a refractory adverse reaction caused by chemotherapy. It was found that in paclitaxel-treated tumor-bearing mice, activation of EGFR signaling in primary sensory dorsal root ganglion (DRG) neurons increased the expression of Dickkopf-1 (DKK1). Targeting DKK1 with specific drugs enhanced the antitumor effects of paclitaxel and alleviate paclitaxel-induced peripheral neuropathy [94].

It’s worth noting that the poor efficacy of chemotherapy in melanoma, despite its known neurotoxicity, presents a fascinating paradox given the pro-tumorigenic role of sensory nerves. Future research should elucidate whether chemotherapy-induced neuropathy creates a dysfunctional, pro-tumorigenic neural microenvironment that counteracts its therapeutic intent. Understanding this specific nerve-tumor crosstalk in melanoma could reveal why chemotherapy fails and suggest novel combination strategies, such as pairing immunotherapy with targeted neural blockade to synergistically overcome resistance, a vulnerability that conventional chemotherapy cannot exploit.

### Adjuvant to cancer immunotherapy

Immunotherapy resistance is common in cutaneous squamous cell carcinoma (cSCC), TNBC, PDAC and melanoma. Cancer-induced sensory nerve injury is a critical mechanism of resistance to anti-PD-1 therapy [95]. This is supported by clinical data showing that perineural invasion (PNI) and nerve injury signatures are enriched in immunotherapy non-responders with cSCC. The core mechanism involves cancer cells directly interacting with and damaging neurons and degrading myelin, which activates an injury program driven by the transcription factor ATF3. This neuronal response establishes a profoundly immunosuppressive TME, characterized by an accumulation of M2-like macrophages (CD163+) and exhausted T cells (CD8 + TIM3+) that renders immune checkpoint blockade ineffective. Crucially, genetically targeting the neuronal injury response by specific knockout Atf3 in nociceptive nerves reverses T cell exhaustion and restores sensitivity to anti-PD-1 therapy, identifying this neuro-immune axis as a targetable vulnerability to overcome treatment resistance.

Bone marrow is the source and reservoir of immune cells, and is widely innervated by sensory nerves [96]. Nociceptor nerves secrete CGRP to drive granulocyte colony-stimulating factor (G-CSF)-induced HSC mobilization via RAMP1 or calcitonin-receptor-like receptor (CALCRL) [97], and also promote myeloid-derived suppressor cells (MDSCs) mobilization in pathological diseases, like stroke [98]. Therefore, in the context of cancer, whether sensory nerves drive MDSCs or other immunosuppressive myeloid cells to migrate into the TME remains to be elucidated. On the other hand, nerve stimulation can also bolster anti-tumor immune response, which is relatively safe and simple to implement [99]. For example, electrical stimulation of the sciatic sensory nerve, combined with ant-PD-L1 immunotherapy, results in superior tumor control compared to monotherapy with immunotherapy in TNBC [46]. In sum, a healthy PNS is essential to hematopoiesis, and regulates immune responses against tumors.

## Conclusion and future directions

The sensory nerve-tumor crosstalk is considered a critical factor in tumor progression, characterized by a mutually beneficial interaction [100]. Tumors or stromal components (CAFs and immune cells) secrete molecules or chemical stimuli to recruit nerve infiltration, and further tame and reprogram nerve types. Sensory nerves in turn accelerate tumorigenesis, metastasis, and even lineage plasticity. In terms of tumor plasticity, it’s unclear how nerves reprogram tumor lineage. It has been confirmed at least that in the SCLC with neuroendocrine phenotype is enriched with glutamatergic sensory neurons [54]. However, whether other neuroendocrine carcinoma like neuroendocrine prostate cancer and neuroendocrine bladder cancer are also innervated by glutamatergic or GABAergic neurons, requires further investigation.

The investigation into the role of sensory innervation in tumorigenesis and malignancy has been largely centered on breast cancer and PDAC. The contribution of sensory nerves to tumorigenesis defies simple classification, as their role, whether pro- or anti-tumor, is highly context-dependent. This functional plasticity is critically influenced by factors such as the tumor’s tissue of origin, the neuron subtype profile, and the specific TME landscape. A prevailing paradigm is that peptidergic sensory nerves, exemplified by CGRP, facilitate tumor advancement. This is challenged by findings in PDAC, where a diminished CGRP-positive neuronal contingent is observed alongside an augmented population of SLIT2-expressing sensory neurons. The functional impact of SLIT2 appears pleiotropic and source-dependent, as it can be secreted by both nerves and the tumor vascular endothelium. In the context of breast cancer, endothelium-derived SLIT2 is specifically responsible for promoting CGRP + nerves innervation and tumor metastasis, a function not shared by the other stromal counterpart. Complicating this narrative are reports suggesting an inhibitory role for SLIT2, which is notably absent from the tumor epithelium. This highlights a critical principle: the biological outcome of SLIT2 signaling is contingent upon the specific cellular source and tumor type. What remains consistent, however, is that SLIT2, regardless of its neuronal or endothelial origin, is fundamentally implicated in malignant progression.

Considering the complex landscape of sensory nerves in TME, further research on their roles across different cancer types is urgently needed. Beyond this, several fundamental questions remain. For instance, further research is urgently needed to elucidate how neuronal inputs influence tumor lineage plasticity and phenotypic evolution. Furthermore, while the nerve-immune axis is established, the precise molecular mechanisms by which neuronal signals orchestrate immune cell trafficking, spatial organization, and function within the TME remain largely unknown. Another fascinating question is whether the location of the neuron’s cell body relative to the tumor impacts its function within the TME.

From a therapeutic perspective, the complexity of nerve-cancer interaction dictates our path forward. The current strategies targeting tumor innervation, including surgical or pharmacological denervation, combinations with chemotherapy or immunotherapy, remain confined to preclinical mouse models. A significant translational gap persists; bridging these promising findings to human clinical trials will require substantial validation to ensure both the safety and efficacy of these novel approaches. The primary challenge is specificity. The goal is to develop strategies that can selectively target nerve fibers inside the TME while leaving the essential nerves in surrounding healthy tissue unharmed. This is crucial for minimizing side effects and creating safe, effective treatments. Progress in these areas holds the key to translating our growing knowledge of cancer neuroscience into real clinical benefits for patients.

## Data Availability

Not applicable.

## Abbreviations

BDNF: Brain-derived neurotrophic factor
CNS: Central nervous system
CAF: Cancer-asscoaited fibroblast
DRG: Dorsal root ganglia
CGRP: Calcitonin gene-related peptide
GC: Gastric cancer
HEV: High endothelial venules
JNC: Jugular-nodose ganglia
MDSC: Myeloid-derived suppressor cells
NGF: Nerve growth factor
NK1R: Neurokinin 1 receptor
NK: Natural killer
OSCC: Oral cavity squamous cell carcinoma
OXPHOS: Oxidative phosphorylation
PNS: Peripheral nerve system
PNI: Perineural invasion
PDAC: Pancreatic ductal adenocarcinoma
RTX: Resiniferatoxin
SP: Substrate P
SCLC: Small cell lung cancer
ssRNA: Single-stranded RNA
SC: Schwann cells
TG: Trigeminal ganglia
TME: Tumor microenvironment
TH: Tyrosine hydroxylase
TNBC: Triple-negative breast cancer
TRPV1: Transient receptor potential vanilloid 1
TLR7: Toll-like receptor 7
TLS: Tertiary lymphoid structures
VG: Vagal ganglia
VEGF: Vascular endothelial growth factor
